# PERSON-CENTERED CARE TO DECREASE LONELINESS AND SOCIAL ISOLATION IN LONG-TERM CARE SETTINGS

**DOI:** 10.1093/geroni/igz038.710

**Published:** 2019-11-08

**Authors:** Megumi Inoue, Mai Hasegawa, Catherine Tompkins, Catherine Donnelly

**Affiliations:** 1 George Mason University, Fairfax, Virginia, United States; 2 JB Line, Inc., Arlington, Massachusetts, United States

## Abstract

The older foreign/immigrant population is predicted to increase in the U.S. As a whole, this population faces greater challenges associated with loneliness and social isolation due to their smaller social networks, language and cultural barriers. Person-centered care should be considered as a potential solution to decrease loneliness and social isolation in long term care settings. In 2017, researchers conducted a thorough examination on the effects of a senior companion program on a Japanese woman with advanced dementia. Results suggest that the client benefitted from the program in regard to her physical wellbeing, emotional wellbeing, language communication and cultural support. Given the support of Japanese companions, the client was able to express her needs and health symptoms effectively and the staff were subsequently able to provide culturally-sensitive care. This study implies that the companion program may benefit other foreign/immigrant older adult populations in reducing social isolation and declining health.

